# New Insights into NF-κB Signaling in Innate Immunity: Focus on Immunometabolic Crosstalks

**DOI:** 10.3390/biology12060776

**Published:** 2023-05-27

**Authors:** Dominga Iacobazzi, Paolo Convertini, Simona Todisco, Anna Santarsiero, Vito Iacobazzi, Vittoria Infantino

**Affiliations:** 1 Bristol Medical School, Translational Health Sciences, University of Bristol, Bristol BS2 8HW, UK; domingaiacobazzi@live.it; 2 Department of Science, University of Basilicata, Viale dell’Ateneo Lucano 10, 85100 Potenza, Italy; paolo.convertini@gmail.com (P.C.); simona.todisco@unibas.it (S.T.); santarsieroanna90@gmail.com (A.S.); 3 Department of Biosciences, Biotechnologies and Biopharmaceutics, University of Bari, Via Orabona 4, 70125 Bari, Italy; vito.iacobazzi1@gmail.com

**Keywords:** NF-κB, innate immunity, macrophages, immunometabolism, gene expression

## Abstract

**Simple Summary:**

NF-κB transcription factors are leading modulators of innate immunity. Recent investigations reveal their interplay with energetic metabolism as a central task of many cell functions, such as immune cell activation. Indeed, requiring energy and metabolites, the defense response must be orchestrated with the cell’s need to save metabolic homeostasis. In this review, new insights about NF-κB function and its crosstalk with energetic metabolism in innate immune cells are discussed in order to improve our knowledge of NF-κB signaling and to better address diagnostic and therapeutic approaches for countless inflammatory diseases.

**Abstract:**

The nuclear factor kappa B (NF-κB) is a family of transcription factors that, beyond their numberless functions in various cell processes, play a pivotal role in regulating immune cell activation. Two main pathways—canonical and non-canonical—are responsible for NF-κB activation and heterodimer translocation into the nucleus. A complex crosstalk between NF-κB signaling and metabolism is emerging in innate immunity. Metabolic enzymes and metabolites regulate NF-κB activity in many cases through post-translational modifications such as acetylation and phosphorylation. On the other hand, NF-κB affects immunometabolic pathways, including the citrate pathway, thereby building an intricate network. In this review, the emerging findings about NF-κB function in innate immunity and the interplay between NF-κB and immunometabolism have been discussed. These outcomes allow for a deeper comprehension of the molecular mechanisms underlying NF-κB function in innate immune cells. Moreover, the new insights are important in order to perceive NF-κB signaling as a potential therapeutic target for inflammatory/immune chronic diseases.

## 1. Introduction

The nuclear factor kappa B (NF-κB) is a pivotal transcription factor involved in the coordination of innate and adaptive immunity, inflammatory responses, and other processes such as cellular differentiation, proliferation, and survival [1]. Years of research have shown that it is expressed in almost all types of cells and tissues, and specific NF-κB binding sites are present in the promoters of hundreds of target genes. The NF-κB system is tightly regulated, and its misregulation is involved in a wide range of diseases, from inflammatory and immune disorders to cancer. It is widely recognized the crucial role played by NF-κB in response to a multitude of stimuli, and therefore it appears clear its involvement in multiple and pathological processes [2].

Nowadays, metabolic changes are widely recognized as a hallmark of a large number of diseases, ranging from chronic inflammatory diseases to cancer. In the last decades, the Warburg effect, a metabolic reprogramming long known as a feature of cancer cells [3], has been observed in activated innate cells such as macrophages and dendritic cells [4,5]. Indeed, countless studies have highlighted the role of metabolic shift as being tightly linked to gene expression reprogramming in order to meet the increased demands for both energy and specific metabolites in cancer as well as in immune cells [6,7,8,9,10,11].

Interestingly, recent observations have broadened the horizons of engagement and the close relationship between the NF-κB system and metabolism, suggesting a close integration and coevolution of nutrient- and pathogen-sensing systems [11,12].

In light of these new findings, after summarizing the most important features of NF-κB signaling and function in innate immunity, this review provides an overview of the emerging link between NF-κB and immunometabolic reprogramming.

## 2. NF-κB Transcription Factor Family

**Figure 1:**
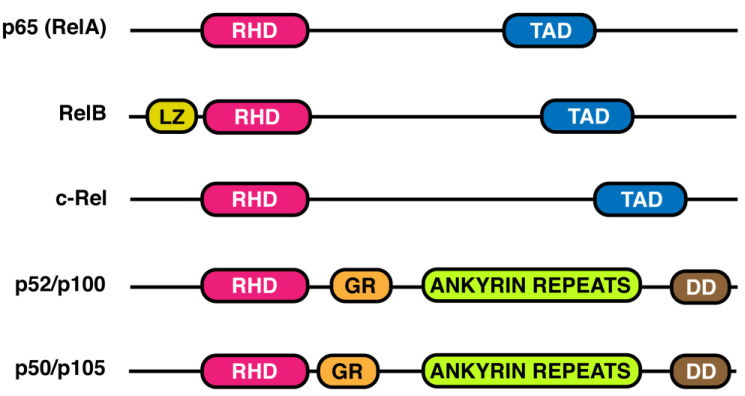
NF-κB family members. Different subunits belonging to the NF-κB family are shown in the figure in relation to the different functional domains. A Rel Homology Domain (RHD), located at the N-terminal region, is common to all members. RelB is the only subunit containing a leucine zipper domain (LZ). A transactivation domain (TAD), at C-terminal, is only present in p65(RelA), RelB, and c-Rel; whereas p52/p100 and p50/p105 share a glycine rich region (GR), ankyrin repeats, and a death domain (DD).

The NF-κB transcription factor family consists of five distinct proteins belonging to the Rel family of proteins, named: p65(RelA), RelB, c-Rel, p105/50 (NF-κB1), and p100/p52 (NF-κB2) [13]. Each member contains a different number of amino acids but shares some common features (Figure 1). The N-terminal contains the Rel Homology Domain (RHD), a sequence of 300 amino acids responsible for dimerization, binding to specific DNA regions, and interaction with IkB regulatory proteins. More detailed crystal studies performed on p50 homo- and p50/p65 heterodimers, the most common NF-κB combination, showed that, within RHD, the N-terminal side is responsible for binding to the consensus sequence on the target genes, whereas the C-terminal mediates the dimerization and interaction with IkBs [14,15,16]. A transactivation domain (TAD), at the C-terminal, is only present in RelA, RelB, and c-Rel (Figure 1). P100 and p105, lacking the TAD domain, share a glycine-rich region (GR), a death domain (DD), and ankyrin repeats (Figure 1), the latter characteristic of the IkB protein family [2]. Interestingly, the presence of a leucine-zipper (LZ) domain makes RelB fully active [17].

As monomers, the NF-κB family members are very unstable and act as activators or repressors only as dimers. RelB mainly dimerizes with p50 and p52, whereas RelA and c-Rel can generate homo- and heterodimers with different members of the family. Thus, NF-κB is a generic name that includes different dimeric proteins generated in different ways. The abundance of a dimer strictly depends on the expression of a monomer in a cell type through a not-yet-clear mechanism, including autoregulation and transcription factors. Apart from RelA, whose control depends on a housekeeping promoter, transcription of other genes encoding NF-κB polypeptides occurs via NF-κB itself, promoting positive feedback in response to cell stimulation [18]. RelA, RelB, and c-Rel proteins are produced in a complete and active form ready to form the dimer, whereas p100 and p105 units need to be processed to a mature form before encountering a dimerization partner [19]. p105 is proteolytically cleaved into p50 by a mechanism that is IKK2-dependent [20] and p100 into p52 via the NIK/IKK1-dependent pathway [21].

Although the number of heterodimers is considerable, the existence and physiological role of all of them have to be demonstrated. The key regulatory mechanisms at the basis of NF-κB dimer formation include the dimerization affinities, the association/dissociation rate constant, the properties of the monomer, the association with a stabilizer, and the degradation process. So far, few studies on these quantitative aspects have been performed. Tsui et al. reported the affinities of RelA/p50 (1–5 nM), p50/p50 (20–50 nM), and RelA/RelA (0.8–15 μM) [22]. Considering the dimer composition of these data, it could be speculated that a dimer generated by a high and a small monomer (i.e., RelA/p52, RelB/p50, etc.) shows the stronger affinity (nM order of magnitude), followed by a dimer composed by two small monomers (i.e., p50/p52, p52/p52). The weaker affinity (μM order of magnitude) should be between dimers composed of two large subunits (i.e., RelB/RelA, RelB/c-Rel, etc.). However, in addition to steady-state affinities, the association/dissociation rate constants should also be considered. The simultaneous presence of different monomers implies that a sort of competition occurs between the monomers to dimerize. For example, in the case of RelA homodimerization and heterodimerization with p50, the high affinity of RelA with p50 significantly reduces the abundance of RelA homodimer. When p50 is significantly reduced, then RelA homodimer formation increases through IkB, which acts as a chaperone or dimer stabilizer [22]. Although induction of NF-κB can be triggered in most cells, it is constitutively active as a nuclear protein in mature B cells, macrophages, neurons, vascular smooth muscle cells, and tumor cells.

## 3. IkB Proteins

NF-κB is normally sequestered in the cytoplasm by a family of inhibitors, named IkB. Thus, association is one of the most important starting features of NF-κB transcription factors. The IKB family is composed of three members, IkBα, IkBβ, and IkBε, which share some common features: two conserved serine residues (DSGXXS) that can be phosphorylated by the IKK subunit and some ankyrin repeats (five to seven). The dimerization of IkB with other NF-κB molecules occurs via their RHDs domain, while their C-terminal ankyrin repeats work as inhibitory proteins [13]. In their inactive state, NF-κB dimers are associated with one of three IkB proteins or with the precursor Rel proteins p100 and p105, which act as inhibitors. This association is essential not only to maintain NF-κB in the cytosol, but it is also crucial for signal responsiveness. In addition, two other atypical IkB proteins, BCL-3 and IkBζ, can be induced upon stimulation and regulate NF-κB, although in a different way. Finally, another IkB protein, named IkBγ, generated as an alternative transcript of the *NF-κB1* gene, was found in mice, but its physiological role is not yet clear [23,24]. Likely, it is believed that IkB proteins associate with a specific subset of NF-κB dimers. For example, IkBα shows higher affinity for p65/p50 complexes than for p65/p65 complexes [25]. RelB binds only to p100; Bcl-3 and IkBζ prefer association with p50 and p52 [26]. However, this aspect needs further investigation.

## 4. Ubiquitin

Ubiquitination is another important hallmark of NF-κB activation. Ubiquitin is produced as a single small protein (8 kDa) synthesized as a precursor for polypeptides needed for post-translational modification. The ubiquitination process involves various subsequent steps: first, ubiquitin is activated by the activating enzyme E1 in an ATP-dependent reaction; then, it is transferred to the ubiquitin conjunction enzyme E2, giving rise to E2-ubiquitin thioester; and finally, by the action of ubiquitin protein ligase E3, E2-ubiquitin thioester is bound to the target protein. Different types of Es enzymes (two E1, tens of E2, and hundreds of E3) have been found, suggesting the involvement of substrate specificity [27]. Ubiquitin can bind substrate as a single monomer (mono-ubiquitination) or multiple units (poly-ubiquitination) through one of the seven lysine residues (K6, K11, K27, K29, K33, K48, and K63). In addition, linear ubiquitination is also possible through the amino-terminal methionine of ubiquitin (the M1 chain). Among the K residues, K48 poly-ubiquitin chains are involved in the signaling and degradation of IkBs, the inhibitors of NF-κB, by the 26S proteasome. Other types of poly-ubiquitin chains are involved in a non-degradative ubiquitination process. These poly-ubiquitins potentiate NF-κB activation by recruiting proteins containing ubiquitin-binding domains (UBDs) [28]. Raighi et al. reported that the UBD domain, composed of the coiled-coil domain and LZ (named CC2-LZ), is crucial for NEMO function and IKK kinase activity [29]. Furthermore, Laplantine et al. showed the formation of a new bipartite ubiquitin-binding domain between the CC2-LZ and the C-teminal LZ domain of NEMO, leading to interaction with K63 ubiquitin chains [30].

As previously reported, a linear ubiquitin assembly (M1) can also be formed. The linear chain is generated by the action of a ligase complex named LUBAC (linear ubiquitin assembly complex), which consists of three subunits: E3 ligase heme-oxided IRP2 ubiquitin ligase-1 (HOIL-1), HOIL-1-interacting protein (HOIP), and SHANK-associated RH domain interactor (SHARPIN) adapter [31,32]. LUBAC binds to NEMO in the IKK complex, inducing oligomerization of the IkB kinase (IKK) complex, which results in the activation of IKK [33,34]. Finally, in the NF-κB pathway, LUBAC interacts with other substrates, such as TNFR1, RIP1, RIP2, IRAK1, IRAK2, MyD88, HOIL-1, and SHARPIN itself, in stimulated macrophages [35,36].

## 5. NF-κB Activation Mechanisms

Two general mechanisms have been ascribed to the NF-κB activation pathway: the canonical and non-canonical, or alternative, pathways. Both are involved in the regulation of immune and inflammatory responses, although via different mechanisms. The canonical pathway needs the NF-κB essential modulator (NEMO)-dependent pathway, while the non-canonical one works through a NEMO-independent pathway.

### 5.1. Canonical Pathway

**Figure 2:**
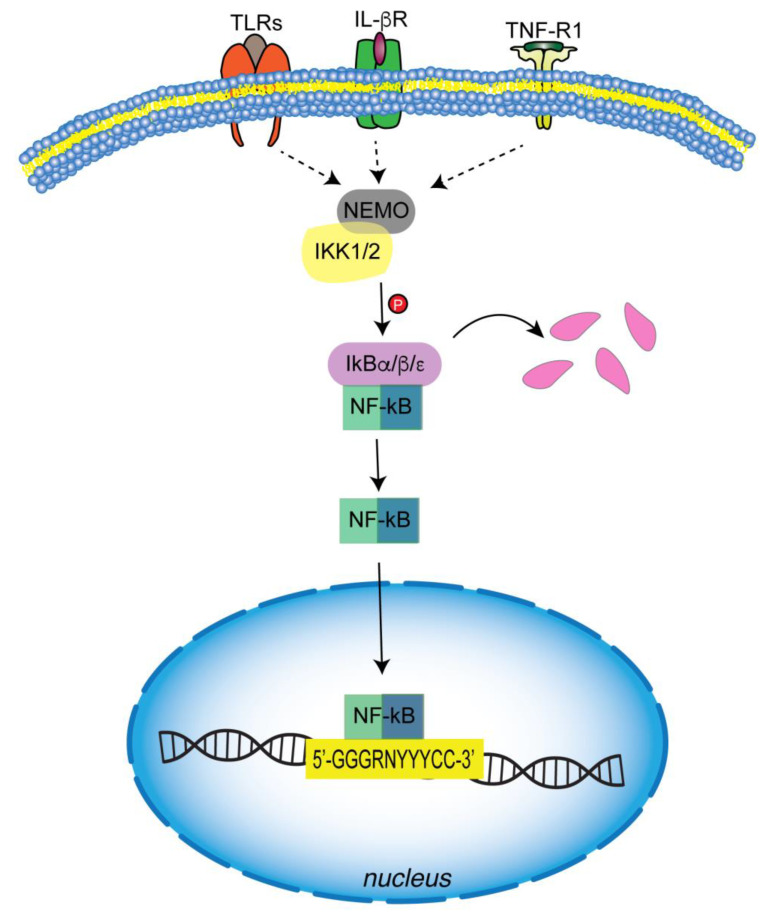
Canonical pathway of NF-κB activation. Several inflammatory signals, such as pathogen-associated molecular patterns (PAMPS) and pro-inflammatory cytokines, activate canonical signaling through a complex containing IKK1/2 and NEMO. IKK1/2-mediated IκBs phosphorylation is a signal for ubiquitination and subsequent proteasomal degradation. Free NFκB dimers enter the nucleus, where they bind consensus DNA sequences and regulate the transcription of target genes.

The initiation of this mechanism is promoted by the action of multiple signals: cytokines, such as TNFα, and interleukin (IL-1β), which bind to their receptors (TNF-R1 and IL-βR receptors); pathogen-associated molecular patterns (PAMPS) and related molecules released by host cells that are recognized by TLRs (toll-like receptors); stress signals, and T and B cell receptors (Figure 2) [1,37]. Signal transduction leads to the activation of a molecular complex through the involvement of kinase enzymes and enzymes involved in ubiquitin chain formation. Among kinases, two active kinases, IKK1 (IKKα) and IKK2 (IKKβ), and a regulatory subunit, NF-κB-essential modulator (NEMO, also known as IKKγ), have been found to be involved in the canonical pathway (Figure 2) [38,39,40]. With regard to the structural features, IKKs contain phosphorylation sites at the N-terminal, a leucine-zipper, responsible for dimerization, in the middle, and a helix-loop-helix (HLH) at the C-terminal, essential for both dimerization and kinase activity [41]. The latter domain of IKKα and IKKβ is responsible for the interaction with IKKγ, the regulatory subunit, which is mediated by a consensus sequence (LDWSWL), named the NEMO-binding domain (NBD) [42,43]. NBD has a helical dimeric structure containing a sequence stretch for interaction with IKK.

IKKs activation is regulated by an autophosphorylation mechanism or by a TGFβ-activated kinase (TAK) complex [44]. IKKs phosphorylate IkBs, allowing the recruitment of the E3ubiquitin ligase SCF/βTRCP and their migration to ubiquitin, where they are degraded via the 26S proteasome. Then NF-κB dimers are released and enter the nucleus, where they bind to the conserved DNA motifs (5′-GGGRNYYYCC-3′; where R = purine, N = any nucleotide, Y = pyrimidine) of target genes for transcriptional regulation of their expression (Figure 2) [27].

Upon activation, the primary effect is the activation of TAD in RelA/p65, and cRel, which dimerize to form both homo- and heterodimers with p50. As reported by Christian et al., phosphorylation of NF-κB subunits impacts their ability to transactivate [45]. For example, phosphorylation of RelA via IKKα is crucial for the negative regulation of proinflammatory gene expression [46].

Further evidence on the importance of IKKα and IKKβ comes from genetic studies in vivo. Severe and morphogenic defects were observed in IKKα-deficient mice, which died prenatally [47]. However, no impairment of NF-κB activation was found in cells lacking IKKα induced by IL-1 or TNFα. The same study highlights the fundamental role played by IKKα in the proliferation and differentiation of epidermal keratinocytes [48]. Furthermore, severe effects were observed in IKKβ-deficient mice: inability of proinflammatory cytokines to activate NF-κB; embryonic lethality within a few days due to severe liver apoptosis in IKKβ-/- mice [49,50,51]. Similarly to IKKβ-/- mice, NEMO-deficient mice undergo death in the embryonic state as a consequence of liver damage [52]. However, female mice survived (the NEMO gene is on the X chromosome) but exhibit skin defects similar to the human disease Incontinentia Pigmenti [53].

### 5.2. Non-Canonical Pathway

**Figure 3:**
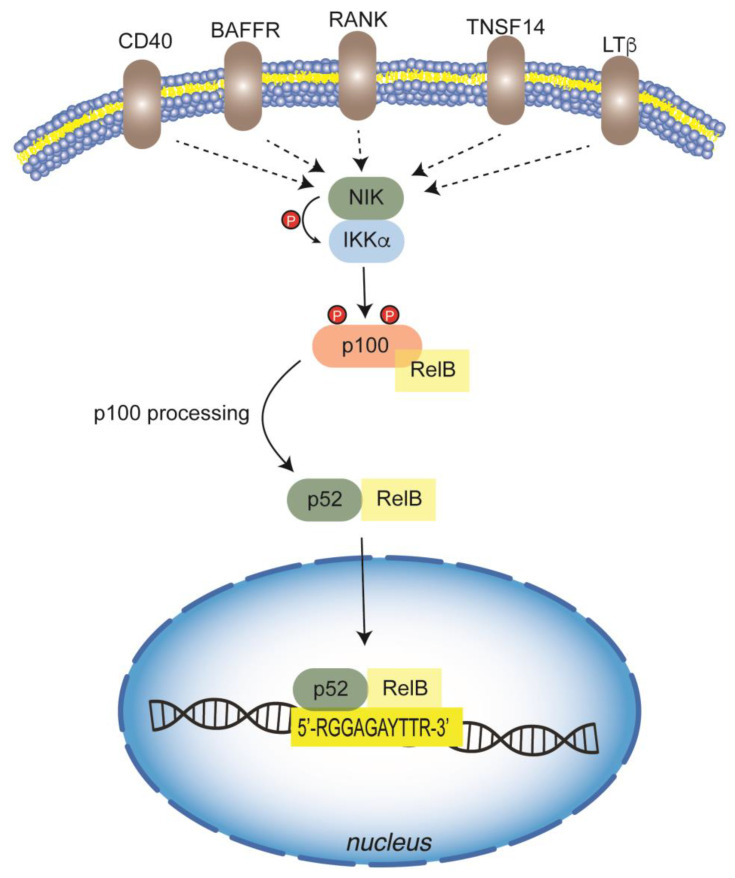
Non-canonical pathway of NF-κB activation. Non-canonical NF-κB pathway is independent of NEMO and relies on another kinase, the NF-κB inducing kinase (NIK), which together with IKKα (IKK1) transduces cell signaling. Multiple membrane receptor-mediated signals activate NIK/IKKα complex, which in turn phosphorylates p100, triggering its processing into p52. The heterodimer p52/RelB can translocate to the nucleus and bind to DNA-responsive elements, thus activating the transcription of target genes.

In the second mechanism, generally named non-canonical, signals are transduced in a NEMO-independent way and involve another kinase, the NF-κB inducing kinase (NIK) and IKKα (Figure 3) [54]. The signal activation is triggered by several receptors: CD40 ligand (CD40), B cell activating factor (BAFF), receptor activator of NF-κB ligand (RANKL), lymphotoxin β (LTβ), TNF-related weaker inducer of apoptosis (WEAK), and tumor necrosis factor superfamily member 14 (TNSF14). Some of these also activate the canonical pathway. After activation, a stabilization and accumulation of NIK occurs, and then it phosphorylates IKKα, which in turn phosphorylates p100. Then, p100 is processed into p52, which binds to RelB to form p52/RelB and other dimers that translocate to the nucleus and bind DNA [21]. Moreover, a significant amount of p100 is also found in an inhibitory complex (IKBδ). Different from other IkBs, IkBδ shows a specific inhibitory action towards RelB and RelA-containing dimers and responds to non-canonical stimuli. NIK knockout failed to induce RelB and RelA in response to LTβR [55].

After phosphorylation, the inhibitory subunit is degraded, whereas the NF-κB dimer is released to enter the nucleus (Figure 3) [55]. It has been reported that the p52/RelB heterodimer can bind to a specific sequence 5′-RGGAGAYTTR-3′ of target genes. However, the crystal structure of the p52/RelB heterodimer is able to bind to the canonical NF-κB responsive element 5′-CGGGAATTCCC-3′ too. Moreover, mutational analysis suggests that the p52/RelB heterodimer would bind to a variety of binding sites and activate countless genes [56]. Therefore, the non-canonical NF-κB pathway represents a mechanism by which specific signaling relies on distinct NF-κB members as well as p52 and RelB in this pathway.

## 6. NF-κB in the Innate Immune Cells

NF-κB plays a pivotal role in pro-inflammatory gene induction and function in both the innate and adaptive immune systems, in which cells such as macrophages, dendritic cells, and neutrophils are actively involved. These cells express *pattern recognition receptors* (PRRs), which recognize different microbial components as (PAMPs). Although, from a structural perspective and in response to different PAPMs and DAMPs (Damage-associated molecular patterns), PRRs differ, they do have similar features in downstream signal transduction. The common pathway involves the canonical NF-κB pathway that induces transcription of pro-inflammatory cytokines, chemokines, and other mediators in different types of innate immune cells (Figure 2). Mediators directly induce inflammation and indirectly promote the differentiation of inflammatory T cells.

Different studies on macrophages highlighted the pro-inflammatory function of NF-κB. As widely acknowledged, macrophages are a large family of innate immune cells present in different tissues that function as a front line in the response against infection [57]. Upon stimulation by PAMPs and DAMPs, monocytes are activated into macrophages, which produce antimicrobial molecules and release cytokines and chemokines. Then, activated macrophages differentiate into phenotypically different types of macrophages, named M1 and M2 [58,59]. These types are characterized on the basis of function and cytokine expression. M1 macrophages produce pro-inflammatory cytokines, including IL-1, IL-6, and TNFα, together with chemokines. Additionally, M1 mobilizes neutrophils, promoting the innate response to pathogens. Furthermore, M1 macrophages stimulate T cell differentiation, such as Th1 and Th17 cells, which successively mediate inflammation [60]. On the contrary, the M2 class of macrophages produces anti-inflammatory cytokines, including IL-10 and IL-13, playing a fundamental role in the resolution of the inflammatory process and in the mediation of wound healing [61]. Toll-like receptors are crucial in the regulation of the M1 polarization process [59]. Lu et al. reported that the LPS/TLR4 signal transduction pathway promotes macrophage differentiation through the involvement of two adapters, MyD88 and TRIF [62]. Both MyD88- and TRIF-dependent pathways are involved in the activation of NF-κB. The MyD88-dependent TLR pathway drives M1 polarization and the expression of pro-inflammatory cytokines. A cascade of activation involving the E3 ubiquitin ligase activity of TRAF6, the ubiquitin-dependent kinase TAKI1, activates IKKs, which phosphorylate the NF-κB inhibitor IkBα, resulting in activation of NF-κB [63]. NF-κB then promotes the induction of a substantial amount of inflammatory genes (TNFα, IL-1β, IL-6, IL-12p40) and cyclooxygenase [59]. The TRIF-dependent TLR signaling pathway induces Type I IFNs and IFN-inducible genes via recruitment of TRAF proteins, especially TRAF3, activation of TANK-binding kinase (TBK1), and IKKε. Upon activation, TBK1 and IKKε phosphorylate the transcription factor IRF3 and induce IFNα and IFNβ [59,64]. Through stimulation of adapter kinase receptor-interfacing protein-1 (RIP1), the TRIF-dependent pathway activates NF-κB [62,65].

## 7. NF-κB and Inflammasome Regulation through Metabolic Signals

Inflammasomes are protein complexes that assemble in response to PAMP and DAMP stimulation. A typical inflammasome consists of ligand-sensing receptors (members of the NLR family), an adapter protein, ASC (apoptosis-associated speck-like protein containing CARD), and pro-caspase 1 [66]. It is considered part of innate immunity against pathogens and also a member of the regulation of intestinal microbiota [67]. Among the inflammasome receptors (NLRP1, NLRP3, NLRC4, and AIM2), NLRP3 is the most extensively studied. Activation necessitates two signals: priming (signal 1) and activation (signal 2). NF-κB is the central mediator in signal 1 inflammasome activation and induces expression of NLRP3 and Pro-IL-1β, both genes containing the NF-κB site in their promoter regions, in response to microbial components (TLR ligands) and cytokines (TNFα, IL-1β). Within the NF-κB system, IKKβ plays a negative role in inflammation activation, likely due to the induction of autophagy, a mechanism by which abnormal proteins and damaged organelles such as mitochondria are degraded [68]. Recruitment of damaged mitochondria is mediated by expression of the p62 autophagy receptor induced by IKK/NF-κB. Consistently, p62-deleted myeloid cells give rise to the accumulation of damaged mitochondria, high production of IL-1β, and endotoxin-induced shock.

In the activation step (signal 2), pro-caspase 1 (pro-Casp 1) is recruited by ASC via CARD-CARD interaction, leading to the assembly of the inflammasome complex. Finally, activated inflammasomes lead to the secretion of proinflammatory cytokines IL-1β and IL-18 and the induction of a gasdermin D-mediated form of cell death known as pyroptosis.

Metabolic stimuli can activate or inhibit the NLRP3 inflammasome. Enzymes, metabolites, and other factors can stimulate inflammasome activation. Glycolytic enzymes, including hexokinase 1 (Hk1), pyruvate kinase M2 (PMK2), and glyceraldehyde-3-phosphate dehydrogenase (GAPDH), regulate the inflammasome [69,70,71]. Inflammasome activation can reprogram PFKFB3 glycolytic metabolism in macrophages through 6-phosphofructo-2-kinase/fructose-2,6-bisphosphatase 3 (PFKFB3) [72]. Furthermore, inhibition of PFKFB3 by 3PO and NLRP3 by MCC950 reduces the LPS- and β-amyloid-induced glycolysis and secretion of IL-1β in bone-derived macrophages (BMDMs) [72].

Among metabolites, β-glucan, a natural polysaccharide, acts as a suppressor of NLRP3 activation by blocking ASC oligomerization and caspase-1 activation in macrophages. Thus, it has been suggested as a candidate drug to treat NLRP3-related disorders [73]. Other metabolites involved in the regulation of NLRP3, such as succinate, β-hydroxybutyrate (BHB), itaconate, 4-octyl-itaconate, and TCA cycle intermediates or derivates, can also inhibit NLRP3 [74,75,76].

The inflammasome is also modulated by saturated fatty acids, polysaturated fatty acids, and phosphatidylinositol [77,78]. Saturated fatty acids stimulate NLRP3 activation in murine macrophages in vitro and the secretion of IL-1β and acute inflammation in vivo [79]. The unsaturated fatty acid oleic acid reverses this phenotype [79]. Interestingly, the transcription factor SREBP2, which plays a master role in cholesterol metabolism, is related to NLRP3 inflammasome activation. Guo and colleagues showed that the SCAP-SREBP2 complex endoplasmic reticulum-to-Golgi translocation was necessary for adequate activation of the NLRP3 inflammasome both in vitro and in vivo [80]. Finally, the NLRP3 acetylation process is implicated in aging-related inflammation and insulin resistance in macrophages. Conversely, deacetylation by Sirt2 prevents inflammation and insulin resistance in aged mice only [81].

## 8. Crosstalk between NF-κB and Metabolism in Innate Immune Cells

In the last few years, an increasing body of literature has suggested an intimate connection between the NF-κB system (immune and inflammatory responses) and metabolism [82]. This link reveals the tight integration and coevolution of nutrient- and pathogen-sensing systems [83].

Before addressing the connection between inflammation, innate immunity, and metabolism through NF-κB, it is worth considering the role of NF-κB in metabolism, independent of the induction of inflammation. NF-κB regulates glycolysis and mitochondrial respiration, depending on the presence of p53 in the cells. In p53+/+ cells, NF-κB/RelA are excluded from mitochondria by p53-mediated disruption of the Mortalin/RelA interaction. Following that, RelA and p53 enter the nucleus, inducing the expression of mitochondrial synthesis of cytochrome c oxidase 2 (SCO2), a subunit of complex IV of the electron transport chain. Induction of SCO2 increases respiration and diminishes glycolysis, together with a decrease in the expression of the glucose transporter (GLUT3). In contrast, in p53-/- cells, glycolysis is induced, whereas OXPHOS is suppressed through inhibition of mitochondrial gene expression by RelA that enters mitochondria via Mortalin-mediated transport. As a consequence of glycolysis induction, the content of cellular glucose increases, resulting in stimulation of NF-κB signaling by promoting O-linked b-N-acetylglucosamine (O-GlcNAc)-linked modification of IKKβ [84,85,86].

Apart from these observations, the most important evidence concerns the crosstalk between the NF-κB system and metabolism in the context of immune and inflammatory responses.

Considering the activation of NF-κB as a central mechanism by which cells respond and coordinate innate immunity and the inflammation response, NF-κB signaling is regulated at different levels. As for the regulation of other pathways, it can be assumed that there is a dual mechanism in which a first response is mediated by canonical and non-canonical activation. These pathways can be defined as “external term regulation” providing a response mediated by external signals produced by extracellular environments. A second mechanism by which metabolic changes sustain NF-κB activation can be hypothesized as “immunometabolic term regulation”.

Both types of regulations guarantee a rapid turn-on and turn-off of NF-κB, underlying the complexity of its regulation.

### 8.1. Metabolic-Linked NF-κB Post-Translational Modifications

**Figure 4:**
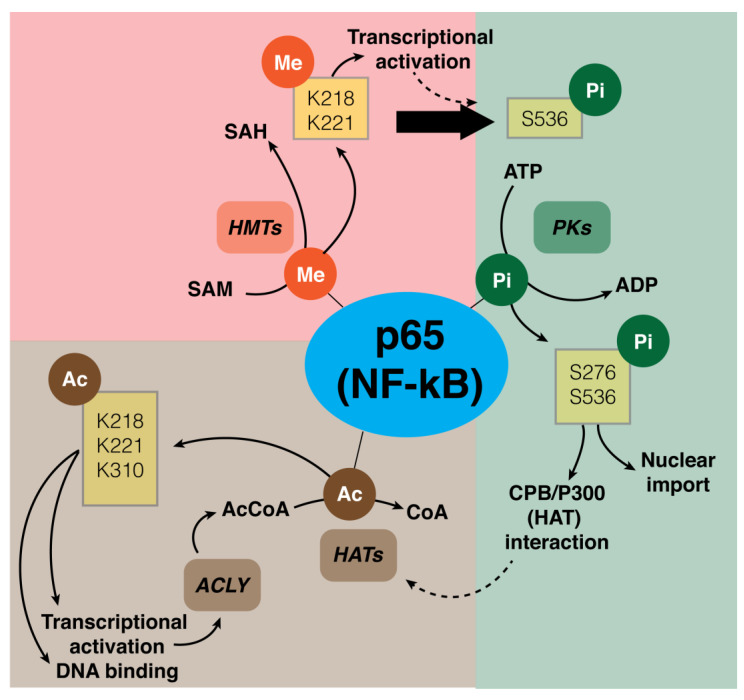
Schematic for the main PTMs of NF-κB (p65 subunit) and their functional activity. Three main PTMs of the p65 subunit are methylation, phosphorylation, or acetylation. Methylation is catalyzed by HMTs that utilize SAM as donor of methyl groups, with formation of SAH. K218 and K221 are two main methylated aminoacidic residues. This modification has the functional effect of increasing the transcriptional activity of NF-κB and, by inhibiting S536 residue dephosphorylation, prolonging the activation. Phosphorylation of p65, catalyzed by PKs, is an important PTM for protein stability and transcriptional activity. Two of the indicated residues, S276 and S536, play a role in nuclear import and protein interaction with CBP/p300. Acetylation of NF-κB is important for DNA binding, and transcriptional activity. HAT catalyzes acetylation by using acetyl-CoA, which is mainly produced by ACLY, one of the NF-κB-regulated genes. Three of the main residues that have been acetylated are K218, K221, and K310. Abbreviation: ME: methyl group, Pi: phosphate group, Ac: acetyl group, HMTs: histone-methyltransferases, SAM: S-adenosyl-L-methionine, SAH: S-adenosyl homocysteine, PKs: protein kinases, HATs: histone acetyl transferases, ACLY: ATP-citrate lyase.

Post-translational modifications (PTMs) are responsible for important mechanisms of complex NF-κB activation and, in particular, of the p65 subunit. Different PTMs of NF-κB are methylation, phosphorylation, and acetylation of the RelA (p65) subunit in the cytosol or nucleus (Figure 4) [87].

In general, NF-κB methylation has several effects on gene expression regulation mediated by different mechanisms [88]. Recent evidence shows that NF-κB is methylated by histone-modifying enzymes that modify histone proteins in addition to non-histone proteins. In particular, NF-κB was found to be methylated on the p65 subunit at levels of six lysines (K37, 218, 221, 310, 314, and 315) by different histone modifying enzymes [88]. Why p65 is methylated on multiple K sites is not clear. It was demonstrated that the methylation of K218 and K221 promotes NF-κB transcriptional activity through interaction with homeodomain finger protein 20 (PHF20) (Figure 4). PHF20 prevents the dephosphorylation of serine 536 of the p65 subunit of NF-κB by the phosphatase PP2A, prolonging the activation of NF-κB [89].

A PTM that has received extensive investigation is represented by p65 phosphorylation, which is important for both protein stability and protein-protein interaction [90]. Four phosphorylated residues (S205, T254, S276 and S281) are localized in the N-terminal of RHD, two residues (S311 and S316) in the C-terminal of RHD, and five residues (T435, S468, T505, S529 and S536) in the C-terminal of TAD. The phosphorylation sites that are more important are S276 and S536. The phosphorylation of S276 and S536 sites causes a conformational change of p65, leading to an increase in interaction with CBP/p300, a histone acetyltransferase (HAT), and an increase in transcriptional activity of p65 (Figure 4) [45,91].

Interestingly, it was suggested that S536 phosphorylation is related to the nuclear import of NF-κB and promotes the proteasomal degradation of p65 (Figure 4). This mechanism limits the NF-κB response in activated macrophages and leads to the resolution of inflammation [92]. The ATP-dependent phosphorylation of the enzyme is strongly regulated and strictly associated with the energetic metabolism to avoid a depletion of cellular ATP. The metabolic interconversion regulates both phosphorylation and dephosphorylation as a regulatory cycle.

Another important PTM is the acetylation of the Rel A (p65) subunit, which has been related to DNA binding interaction, transcriptional activity, and protein interaction [93]. The main acetylated identified lysines include K122, K123, K218, K221, K310, K314, and K315. In particular, K221acetylation enhances NF-κB’s ability to bind to the DNA, and the acetylation of both K218 and K221 regulates the interaction with IκBα, prolonging the NF-κB response [93]. Furthermore, the acetylation of K310 increases the transcriptional activity of NF-κB, while K122 and K123 residue acetylation is fundamental for facilitating the termination of the NF-κB response (Figure 4) [94].

### 8.2. NF-κB and Immunometabolic Interplay

**Figure 5:**
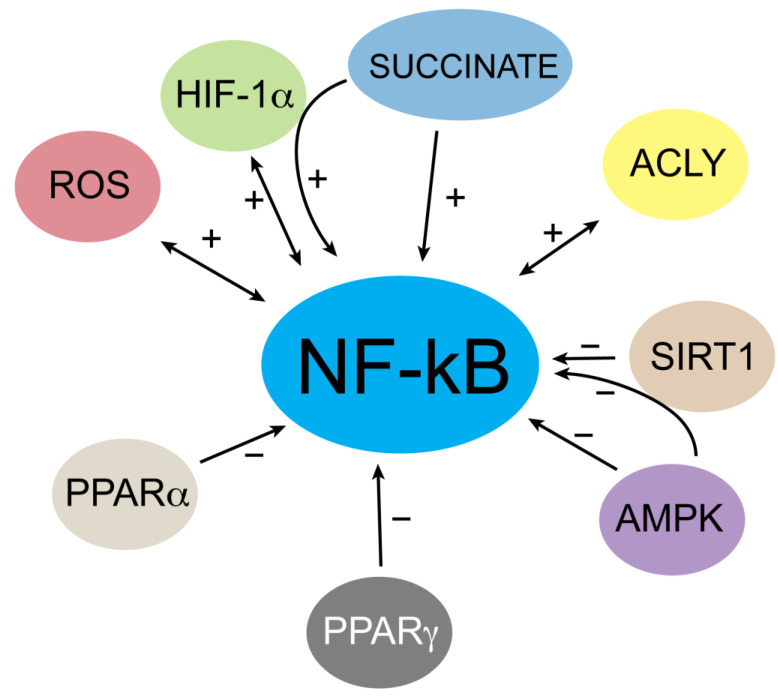
Summary of the interplay between NF-κB and metabolic signals in innate immune cells. AMP-activated protein kinase (AMPK) negatively affects NF-κB activity dependent or independent of SIRT1. Through the hypoxia-inducible factor 1 subunit α (HIF-1α), increased levels of succinate act positively on NF-κB in M1 macrophages. Moreover, a solid crosstalk based on mutual regulation between HIF-1α and NF-κB occurs in innate immunity. NAD^+^-dependent SIRT1 lowers NF-κB transcriptional activity by p65 subunit deacetylation. ATP citrate lyase (ACLY) fosters p65 subunit acetylation, thus inducing NF-κB activation, which in turn upregulates *ACLY* human gene together with countless proinflammatory genes in M1 macrophages. Both the peroxisome proliferator-activated receptors PPARα and PPARγ suppress NF-κB signaling. Finally, a positive interaction takes place between ROS and NF-κB.

Considering the central role of NF-κB in inflammation and innate immunity [95], a recent interesting regulation loop occurring via NF-κB acetylation has emerged. It has been demonstrated that the immunometabolic enzyme ATP citrate lyase (ACLY) controls NF-κB p65 subunit activation, supporting the relevance of immunometabolism in the regulation of immune cell function. Indeed, in macrophages activated by LPS as well as lipoteichoic acid (LTA), ACLY has been found to be upregulated [11,96], and its acetylation at the level of both K662 and K665 residues controls ACLY nuclear translocation. In the nucleus, ACLY-derived acetyl-CoA is used for NF-κB p65 subunit acetylation, increasing its transcriptional activity. Interestingly, NF-κB controls the promoter activity of *ACLY* together with the *SLC25A1* human gene [97], encoding the mitochondrial citrate transporter CIC. In this way, the macrophage response is sustained by a metabolic reprogramming in which the upregulation and activation of the citrate pathway, via *ACLY* and *SLC25A1*, leads to nuclear migration of ACLY. NF-κB activation mediated by ACLY fosters the transcription of many pro-inflammatory genes, in turn promoting the inflammatory cascade (Figure 5). These new findings allow for a greater comprehension of the mechanism by which vegetables such as red wine or specific plant-derived extracts [98,99] affect NF-κB activity in order to consider NF-κB as a potential therapeutic target for inflammatory diseases.

The balance of NF-κB-mediated immune response is under the control of many other metabolic regulators, for example AMPK, SIRT1, HIF, PPARα and PPARγ.

AMPK (AMP-activated protein kinase) is an energy sensor that promotes catabolism through the induction of mitochondrial biogenesis and oxidative metabolism gene expression. This oxidative metabolism is typical of quiescent or anti-inflammatory cells such as resting or M2 macrophages. AMPK inhibits the NF-κB signaling pathway by acting in an indirect manner via mediators such as SIRT1 or peroxisome proliferator-activated receptor γ co-activator 1α (PGC-1α) [100] (Figure 5). Remarkably, it has been demonstrated that SIRT1 (nicotinamide adenosine dinucleotide-dependent histone deacetylase) is responsible for lysine 310 deacetylation of the p65 subunit, thus reducing the transcriptional activity of NF-κB and inhibiting the inflammatory response [101,102]. Notably, SIRT1 activity is affected by intracellular NAD^+^ levels, strengthening the interplay between metabolism and immune response.

NF-κB activation is also affected by hypoxia in different manners, one of which involves the transcriptional factor HIF (Hypoxia-Inducible Factor). Interestingly, a feedback loop involves the crosstalk between HIF and NF-κB, in which NF-κB has been found to be a direct modulator of HIF-1α in inflammation by controlling HIF-1α transcription (Figure 5). Similarly, HIF-1α activation fosters NF-κB-mediated proinflammatory gene expression in activated innate immune cells such as macrophages and neutrophils, although it has also been reported that HIF-1α can inhibit NF-κB activity in cancer cells [103,104].

Peroxisome proliferator-activated receptor-α (PPARα) is known to positively regulate the anti-inflammatory response by indirectly inducing fatty acid β-oxidation [105]. Recent findings show that PPARα directly affects NF-κB signaling in macrophages. Particularly, PPARα inhibits NF-κB by altering in vitro macrophage polarization, thus showing an anti-inflammatory effect [106].

The repression of p65 transactivation is one of the ways by which PPARα interferes with the NF-κB pathway. This mechanism occurs via a direct interaction between PPARα and the p65 subunit. Another mechanism involves the PPARα-mediated activation of anti-oxidant enzymes, which reduces oxidative stress and, as a result, the activation of NF-κB. Finally, PPARα induces IkBα expression that affects the binding of NF-κB to DNA [107].

In addition, PPARγ, another member of peroxisome proliferator-activated receptors, has been found to regulate the polarization of alveolar macrophages. PPARγ induces a switch from the M1 to M2 macrophage phenotype through the decrease of nitric oxide production and the inhibition of NF-κB. In fact, PPARγ agonist treatment improves macrophage polarization by inhibiting NF-κB p65 subunit phosphorylation in alveolar macrophages [108].

Pro-inflammatory conditions associated with NF-κB activation are characterized by an increase in ROS levels, with particular reference to superoxide anion, an element of inflammasome activation too. For example, ROS are essential to kill pathogens in the respiratory burst, in which NADPH oxidase, together with inducible nitric oxide synthase (iNOS), superoxide dismutase (SOD), and myeloperoxidase, is involved in the production of HClO^−^, a potent antimicrobial compound [109]. NF-κB is responsible for the transcriptional activation of many target genes related to the pro-inflammatory response as well as all the genes mentioned above to induce ROS production. It is well known that high concentrations of ROS can be toxic to cells [110]. For this reason, ROS may help modify the NF-κB-mediated response to support cellular survival [111]. Thus, NF-κB can be viewed in this context as a cell fate checkpoint, and, also in this case, a feedback cycle exists in which ROS levels are regulated and in turn regulate the NF-κB signaling pathway (Figure 5).

Indeed, innate immune cell activation requires ROS production, but ROS levels must be tightly regulated because high levels trigger cell apoptosis. Therefore, when ROS levels increase, NF-κB induces the transcription of antioxidant genes as well as manganese superoxide dismutase (MnSOD, or SOD2) and thioredoxins [112], glutathione S-transferase pi (GST-pi) [113], and glutathione peroxidase-1 (Gpx1) [114].

The crosstalk between NF-κB signaling and ROS production also results from a deep metabolic reprogramming in innate immune cells such as M1 and M2 macrophages. In general, M1 macrophagic metabolism is characterized by a high glycolytic flux, an increase in the pentose phosphate pathway (PPP) and fatty acid biosynthesis, and a reduction in the TCA cycle and oxidative phosphorylation. PPP contributes to NADPH production for ROS and NO biosynthesis [115], whereas fatty acids contribute to the pro-inflammatory response. The TCA cycle displays two break points at the citrate and succinate levels, which causes an accumulation of these two metabolites in M1 macrophages. Citrate supports the pro-inflammatory response by sustaining the biosynthesis of fatty acids and pro-inflammatory molecules, among which are the superoxide anion and nitric oxide [116,117]. The accumulation of succinate stabilizes HIF1α by inhibiting prolyl hydroxylases (PHDs) [118], in a hypoxia-independent manner that could turn into an indirect regulation of the NF-κB pathway. Finally, the electron flow direction in oxidative phosphorylation is reversed, leading to high ROS production [119]. On the contrary, in M2 macrophages, oxidative phosphorylation and the TCA cycle increase, as does glutamine metabolism for M2 activation.

Collectively, these recent investigations point up how immunometabolism relies on NF-κB to drive innate immune cell activation and inflammation but also to regulate different cell phenotypes. A thorough elucidation of the metabolic activators and effectors of NF-κB signaling could translate into an effective approach for potential therapeutic NF-κB targeting in inflammatory diseases.

## 9. Concluding Remarks

The department’s investigation in the last decades has established the fundamental role played by the NF-κB system in controlling metabolic networks that shape other biological aspects such as cell differentiation, energy homeostasis, pathologies, and cancer. Due to these multifaced links, understanding the complex mechanism underlying NF-κB regulation remains an important goal to facilitate intervention on specific steps on the NF-κB pathways, paving the way for more appropriate therapies. Here we have focused on the new insights into NF-κB function in innate immunity and the crosstalk between NF-κB signaling and metabolism.

## Data Availability

Not applicable.
